# Voluntary Modulation of Anterior Cingulate Response to Negative Feedback

**DOI:** 10.1371/journal.pone.0107322

**Published:** 2014-11-06

**Authors:** Matthew S. Shane, Christina R. Weywadt

**Affiliations:** 1 University of Ontario Institute of Technology, Oshawa, Ontario, Canada; 2 The Mind Research Network, Albuquerque, New Mexico, United States of America; 3 University of New Mexico, Albuquerque, New Mexico, United States of America; University Of Cambridge, United Kingdom

## Abstract

Anterior cingulate and medial frontal cortex (dACC/mFC) response to negative feedback represents the actions of a generalized error-monitoring system critical for the management of goal-directed behavior. Magnitude of dACC/mFC response to negative feedback correlates with levels of post-feedback behavioral change, and with proficiency of operant learning processes. With this in mind, it follows that an ability to alter dACC/mFC response to negative feedback may lead to representative changes in operant learning proficiency. To this end, the present study investigated the extent to which healthy individuals would show modulation of their dACC/mFC response when instructed to try to either maximize or minimize their neural response to the presentation of contingent negative feedback. Participants performed multiple runs of a standard time-estimation task, during which they received feedback regarding their ability to accurately estimate a one-second duration. On *Watch* runs, participants were simply instructed to try to estimate as closely as possible the one second duration. On *Increase* and *Decrease* runs, participants performed the same task, but were instructed to “try to increase [decrease] their brain's response every time they received negative feedback”. Results indicated that participants showed changes in dACC/mFC response under these differing instructional conditions: dACC/mFC activity following negative feedback was higher in the *Increase* condition, and dACC activity trended lower in the *Decrease* condition, compared to the *Watch* condition. Moreover, dACC activity correlated with post-feedback performance adjustments, and these adjustments were highest in the *Increase* condition. Potential implications for neuromodulation and facilitated learning are discussed.

## Introduction

The ability to adaptively manage goal-directed behavior requires a consistent monitoring for, and adjustment to, indication of error in performed actions. To this end, electrophysiological and functional magnetic resonance imaging (fMRI) work have converged to demonstrate important neural signatures that appear sensitive to the receipt of feedback indicating goal-directed error [1]–[4]. This work originated with the identification of the feedback error-related negativity (fERN) – a unique electrophysiological signature that occurs reliably between 250–400 ms following the receipt of feedback indicating goal-directed error [1]–[2]. Source localization work has subsequently identified the likely source of this activity to the dorsal anterior cingulate cortex (dACC) and/or adjacent medial frontal cortex (mFC), and convergent fMRI work has confirmed a central role for dACC/mFC in the detection of negative feedback [3]–[4].Contemporary models of reinforcement learning now stress that this dACC/mFC response likely reflects the generation of a critical neural reward-prediction error signal that indicates when outcomes do not occur as expected [5]. Indeed, several studies have reported that individuals with greater amplitude fERNs and/or increased dACC/mFC response to negative feedback show more adaptive post-feedback performance adjustment and/or greater operant learning proficiency [6]–[8]. This has led researchers to posit that dACC/mFC response to negative feedback represents not only a feedback detection system, but also a critical component for the adaptive selection and guidance of subsequent corrective behavior [9]–[10].

A burgeoning area of work has become interested in the extent to which brain activity may be responsive to adaptive modulation. Emerging technologies including transcranial direct current stimulation (TDCS) [11], transcranial magnetic stimulation (TMS) [12], and real-time functional magnetic resonance imaging (rt-fMRI) [13] have all shown potential for facilitating changes in specific neural firing patterns. More basic even, is complimentary work on “emotion regulation”, which has demonstrated that participants are capable of engendering changes in neural responses, simply through voluntarily-initiating up- or down-modulation of reactivity to presented stimuli [14]–[16]. In a standard emotion regulation paradigm, participants are shown a variety of emotionally-valent pictorial stimuli, and are simply asked to try to intentionally increase or decrease their responsivity during stimulus processing. Evidence suggests that participants are quite capable of voluntarily modulating their neural responses in this fashion. Moreover, several studies have reported important relationships between voluntarily-induced modulation of neural responses, and subsequent changes in either behavior [17]–[18] or experience [19]–[20]. Thus, the ability to voluntarily modulate neural reactivity may be more than academic, and may offer a variety of useful real-world applications.

One such application could be the ability to foster improved sensitivity to information indicating error in goal-directed behavior. Increased sensitivity to error could promote improvements in cognitive control and/or operant learning proficiency. Decreased sensitivity to error could, in turn, prove adaptive for individuals with characteristic hypersensitivity to error (e.g., high-anxious populations) [21]. With this in mind, the present study sought to evaluate the extent to which participants could modulate their neural responses following the receipt of negative feedback within a standard time-estimation task (within which they received veridical positive or negative feedback indicating the accuracy of their attempts to estimate a one-second duration). Closely mirroring the technique employed in studies on emotion-regulation, each participant performed this task under three instructional conditions. In a *Watch* condition, participants performed the task normally, with standard instructions to estimate the one-second duration as accurately as possible. In *Increase* and *Decrease* conditions, participants were instructed to perform the same task, but to “try to increase [decrease] your brain's response” as much as possible following the receipt of negative feedback. In this way, the study afforded a careful within-subject evaluation of the extent to which individuals could voluntarily modulate their neural responses to the presentation of contingent negative feedback. Of particular interest were changes in participants' dACC/mFC response, given its well-established involvement in error-detection and action-monitoring. In addition, we sought to evaluate the relationship between dACC/mFC activity and post-feedback performance adjustments by evaluating changes in estimation attempts from trial *n* to trial *n*+1. We hypothesized that our sample of healthy individuals would show changes in dACC/mFC response following negative feedback in the direction instructed. Moreover, we hypothesized that these changes in neural response would be related to the magnitude of participants' post-feedback performance adjustments.

## Method

### Participants

Eighteen healthy individuals (8 females) were recruited through advertisements posted on The University of New Mexico campus. Age ranged from 18 to 44 (*M* = 25.00, *SD* = 6.27).

### Time Estimation Task

**Figure 1:**
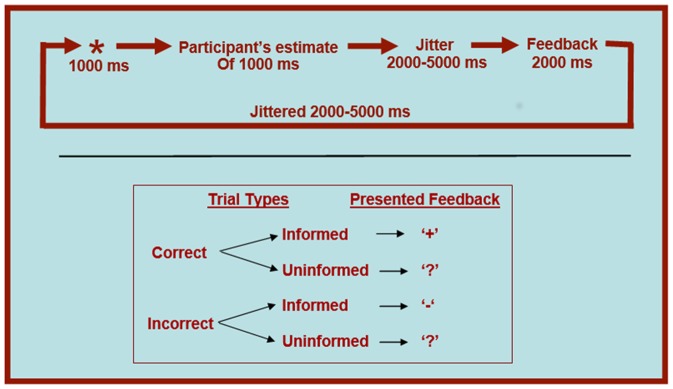
Task details and trial type organization.

The time estimation task (depicted graphically in Figure 1) required that participants attempt to estimate as accurately as possible a one-second duration. Each participant performed 5 practice trials, followed by 60 experimental trials, all of which were similarly designed, and modeled after previous work [4]. Each trial began with a large asterisk presented on-screen for 1000 ms. Participants were informed that they should wait for the asterisk to disappear, and then try to press a button with their right index finger exactly 1000 ms after the asterisk's offset. Following their button press was a randomly jittered interval (2000 ms, 3500 ms, or 5000 ms) to improve deconvolution from the standard hemodynamic response curve. Finally, participants received feedback regarding the accuracy of their estimate attempt. This feedback came in one of two varieties: on *informative feedback trials*, participants received either a plus sign (‘+’) or a minus sign (‘-‘), which indicated whether their estimate was accurate or inaccurate on that trial. On *uninformative feedback trials*, participants received only a question mark (‘?’), regardless of whether their estimate was accurate or inaccurate. These question mark trials were presented on exactly 50% of all accurate trials and 50% of all inaccurate trials. Thus four different trial types were possible (*Informed-Accurate, Informed-Inaccurate, Uninformed-Accurate, Uninformed-Inaccurate)*, and each occurred with near-equal frequency. The uninformative trials may appear cumbersome, but constituted a critical component of the study design: because participants' actual estimation accuracy could be matched across informative and uninformative trials, a direct comparison of these trials afforded a careful control for well-established effects of outcome anticipation on neural responses to feedback stimuli [5], [22] (see Data Analytic Strategy section below for additional discussion within the present paper). Following a second jittered interval (2000 ms, 3500 ms, or 5000 ms) the next trial began.

Participants' estimates were deemed accurate if they fell within a specified window surrounding 1000 ms. The initial window was set at ±250 ms; thus time estimates between 750 ms and 1250 ms received accurate feedback, and estimates that fell outside this window received inaccurate feedback. To ensure that participants received an equal number of accurate/inaccurate feedback trials, an adaptive algorithm was employed, such that the window of accuracy increased by 30 ms following each accurate estimate, and decreased by 30 ms following each inaccurate estimate. As in previous research, this algorithm allowed for the nearly equal presentation of positive (51.5% of all trials) and negative (48.5% of all trials) feedback trials in a manner that was undetectable by participants.

### Procedure

After providing informed consent, participants completed 5 practice trials to familiarize themselves with the task. Following these practice trials, participants performed six separate 60-trial runs of the time-estimation task described above (two *Watch* runs, two *Increase* runs, two *Decrease* runs). Thus, participants completed 120 *Watch* trials, 120 *Increase* trials, and 120 *Decrease* trials of the task over the course of a single one-hour MRI session. All participants performed the two *Watch* runs first, to establish their baseline neural response to the positive and negative feedback, followed by the *Increase* and *Decrease* runs, which were presented in counter-balanced order. On *Watch* runs, participants were instructed to press a button as soon as they believed a one-second duration had expired following the offset of the presented asterisk. They were informed that they would receive accuracy/inaccuracy feedback, however no additional instructions were provided regarding how to process that feedback. On *Increase* and *Decrease* runs, participants were again instructed to estimate this one-second duration, but were explicitly instructed to “try to increase [decrease] your brain's response every time you receive negative feedback”. Participants were not provided with instructions for how to accomplish this neuromodulation; rather, they were told that the intent of the study was to determine whether they could accomplish this by their own devices.

### Ethics Statement

All procedures were approved by The University of New Mexico Internal Review Board, and were in accordance with the provisions of the World Medical Association Declaration of Helsinki. All participants provided written informed consent to participate in the study.

### Data Acquisition

All fMRI data collection was performed using a Siemens TIM Trio 3 Tesla MRI system. Images were presented with a JVC DLA Multimedia projector (Model DLA-SX200-NLG) using E-Prime 2.0 software [23]. Thirty-two axial slices covering the whole brain (3.5 mm) were collected using a gradient echo-planar pulse sequence (TR = 2000 ms, TE 29 ms, FA = 65, FOV: 24×24 cm, 64×64 matrix, 3.44 mm×3.44 mm in plane resolution, flip angle: 75°). All preprocessing and GLM-based statistical analyses of data were carried out using Statistical Parametric Mapping 5 (SPM5) as described below.

Functional images were reconstructed offline and reoriented to approximately the anterior commissure/posterior commissure (AC/PC) plane. Functional image runs were motion corrected using an algorithm unbiased by local signal changes (INRIAlign) [24] as implemented in SPM5. No participants showed head movements in excess of 5 mm, and thus all 18 participants were retained for the analyses reported below. A mean functional image volume was constructed for each run from the realigned image volumes. The mean EPI image was normalized to the EPI template. The spatial transformation into standard MNI space was determined using a tailored algorithm with both linear and nonlinear components [25]. The normalization parameters determined for the mean functional volume were then applied to the corresponding functional image volumes for each participant. The normalized functional images were smoothed with a 9 mm full width at half-maximum (FWHM) Gaussian filter. A high-pass filter (cutoff period 116 hz) was applied to remove any low-frequency confounds. A latency variation amplitude-correction method was used to provide a more accurate estimate of hemodynamic response for each condition [26].

### Data Analysis

Individual participant data was analyzed using a mixed-effects event-related model in SPM5. The asterisk cue, the participant's response (accurate, inaccurate), and the feedback presentation (*Informed-Accurate, Uninformed-Accurate, Informed-Inaccurate, Uninformed-Inaccurate*) were each modeled as separate events. The primary event of interest, feedback presentation, was modeled with a standard hemodynamic response function with 2 s duration. Contrast images corresponding to *Informative-Accurate, Informative-Inaccurate, Uninformative-Accurate and Uninformative-Inaccurate* trials were computed separately for each of the *Watch, Increase* and *Decrease* conditions, and compared against each condition's implicit baseline using the general linear model.

Group analyses utilized a random effects ‘flexible factorial' approach in SPM5 to create a 3 (Instruction: *Watch, Increase, Decrease*) ×4 (Feedback: *Informed-Accurate, Informed-Inaccurate, Uninformed-Accurate, Uninformed-Inaccurate*) within-group ANOVA at the second level. Evaluation of higher-order main effects and interactions were followed by *t-*contrasts, guided by *a priori* hypotheses, which focused on comparing the *Informed-Inaccurate > Uninformed-Inaccurate* contrast across each of the *Watch, Increase* and *Decrease* conditions. Activity in the *Watch* condition was used as a representation of participants' neural responses upon receipt of *Informative-Inaccurate* feedback under standard “passive-viewing” conditions. Activity in the *Increase* and *Decrease* conditions, in turn, was used as a representation of the extent to which participants could follow the instruction to voluntarily increase or decrease their neural activity following receipt of the same *Informed-Inaccurate* feedback. Analyses thus evaluated *Informed-Inaccurate > Uninformed-Inaccurate* BOLD response within each of the three instruction conditions, and also evaluated the extent to which *Informed-Inaccurate* activity in the *Increase* and *Decrease* conditions differed from activity in the *Watch* condition.

For authenticity, we also report results for the *Informed-Inaccurate > Informed-Accurate* contrast; however, we primarily focus on the *Informed-Inaccurate > Uninformed-Inaccurate* contrast because we believe this contrast affords a more careful control for the participants' *actual* estimation accuracy. Increased control of this nature may generally be viewed as advantageous, but may be particularly important in the present context, given the well-established reciprocality of neural responses during the expectation and presentation phases of negative feedback processing [5], [22]. Because of this reciprocality, neural responses during the presentation-phase of negative feedback processing have been shown to attenuate if participants have formed a previous expectation that such feedback is likely. In the *Informed-Inaccurate > Informed-Accurate* contrast the accuracy of participants' estimates (and, by proxy, their expectation of accurate or inaccurate feedback) is likely to vary. In contrast, the *Informed-Inaccurate > Uninformed-Inaccurate* contrast afforded a careful matching of participants' actual estimation accuracy, and thus minimized the likelihood that expectation-related effects would complicate the data.

All data were intensity-thresholded at p<.001, with a cluster-size correction undertaken via AlphaSim to equate to a family wise error (FWE) rate of *p*<.05 (*k* = 19). In addition, two 10 mm regions of interest (ROIs) spheres were constructed, within dACC (central coordinate: *x* = 9, *y* = 30, *z* = 27) and mFC (central coordinate: *x* = 6, *y* = 15, *z* = 57), to allow for optimal evaluation of activity within regions with primary involvement in error-monitoring. Coordinates for these ROIs were arrived at by averaging coordinates obtained through an instructed sampling of the relevant literature on error-monitoring responses to negative feedback [27], [28], and were thresholded at *p*<.05, FWE-svc.

#### Voluntarily-induced modulation and post-feedback performance adjustments

To evaluate the extent to which voluntarily-induced changes in neural response to negative feedback would influence performance adjustments on the following trial, we calculated ‘estimation change scores' for each participant on a trial-by-trial basis, by calculating the absolute value of (participants' estimation time on trial *n*) - (participant's estimation time on trial *n+1*). For instance, if a participant's time estimates on successive trials were: 1250 ms, 950 ms, and 1400 ms, then two change scores could be calculated as follows:


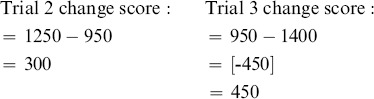


Note that by using absolute values, change scores were not sensitive to the direction of change. This was deemed appropriate given that the negative feedback did not provide participants with directional information.

Three mean change scores were calculated, representing the extent to which participants adjusted their estimation attempt on trials following *Informed-Inaccurate* feedback in each of the *Watch, Increase* and *Decrease* instruction conditions. These estimation change scores were then interrogated in two complimentary ways. First, change scores were entered into SPSS and evaluated via one-way ANOVA to identify behavioral differences in adjustment magnitude across the three instruction conditions. Second, estimation change scores were entered as a first-level parametric modulator (tied to the feedback presentation) in SPM5, and then interrogated via a 3 (Instructions: *Watch, Increase, Decrease*) ×4 (Feedback: *Informed-Accurate, Informed-Inaccurate, Uninformed-Accurate, Uninformed-Inaccurate*) “flexible-factorial” ANOVA, to afford whole-brain (*p*<.05, FWE, cluster-corrected via AlphaSim (*k* = 19)) and ROI (*p*<.05, FWE-svc) evaluations of neural activity that varied with estimation change scores on a trial-by-trial basis. As before, higher-level main effect and interaction analyses were followed by planned comparisons capable of targeting instruction-related differences across each of the *Informed-Inaccurate > Uninformed-Inaccurate* contrast images.

## Results

### Behavioral Results

After evaluation with box and whisper plots, trials during which estimation attempts were greater than 3654 ms (3 standard deviations above the mean; 1.7% of all trials) were removed from the dataset. One participant showed particularly high inaccuracy (16.7% of their estimates fell above the exclusion threshold); thus their data was excluded from the analyses reported below. For the remaining 17 participants, the adaptive algorithm was highly successful in balancing the presentation of accurate/inaccurate feedback. Across all participants, 51.5% of all trials (62 trials/participant) were scored as accurate, and 48.5% (58 trials/participant) of all trials were scored as inaccurate.

Mean estimation time across all trials was 1125 ms (SD = 144 ms; range  = 901–1354 ms). These estimation times were analyzed within a 3 (Instruction: *Watch, Increase, Decrease*) ×4 (Feedback: *Informed-Accurate, Informed- Inaccurate, Uninformed-Inaccurate*) repeated-measures ANOVA, which revealed a main effect of Feedback, *F* = 19.8, η2 = .553, *p*<.001, but no Instruction, *F* = 1.65, *p* >.10, or Feedback x Instruction, *F* = 1.18, *p* >.10, effects. The main effect of Feedback followed anticipated patterns: estimation attempts were shorter and less variable on accurate (*Informed-Accurate*: *M* = 1043 ms; *SD* = 80 ms; range  = 901–1178 ms; *Uninformed-Accurate*: *M* = 1043 ms; *SD* = 76 ms; range  = 922–1159 ms) than on inaccurate (*Informed-Inaccurate*: *M* = 1215 ms; *SD* = 208 ms; range  = 896–1524 ms; *Uninformed-Inaccurate*: *M* = 1199 ms; *SD* = 223 ms; range  = 851–1575 ms) trials (mean difference: *t*(16)  = 4.78, *p*<.001; variance difference: *t*(16)  = 6.67, *p*<.05).

### Neuroimaging Results

#### Analysis of variance brain activation maps

**Figure 2:**
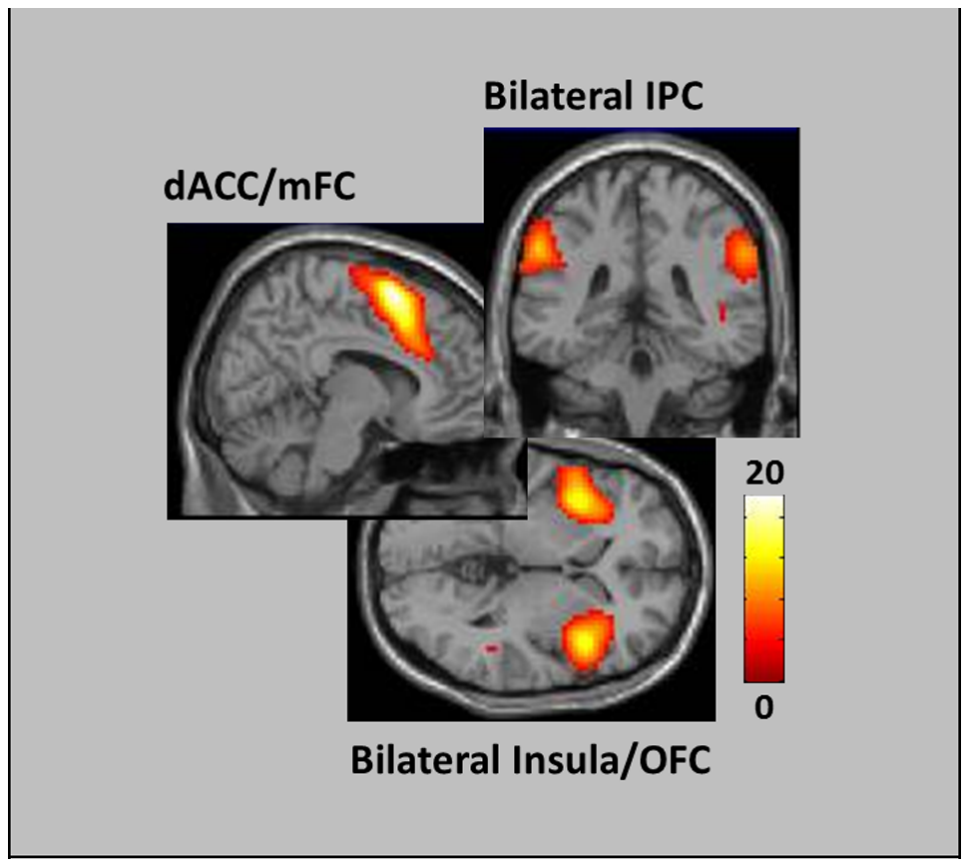
Clusters showing significant changes in the 4 (Feedback) ×3 (Instruction) Interaction.

Evaluation of the 3 (Feedback: *Watch, Increase, Decrease*) ×4 (Instruction: *Informed-Accurate, Informed-Inaccurate, Uninformed-Accurate, Uninformed-Inaccurate*) ANOVA revealed a significant main effect of Feedback within various regions including dACC/mFC, bilateral insula, bilateral orbitofrontal cortex, and bilateral inferior parietal cortex (see Figure S1). Subsequent paired *t-*tests indicated that this main effect was carried by the *Informed-Inaccurate* condition, which showed increased dACC/mFC activity compared to the other three feedback types (all *t*s >7.68). A significant main effect of Instruction was also revealed, within distinct clusters that included bilateral putamen and bilateral dorsal/ventral attentional streams (see Figure S2). These main effects were modulated by a significant Feedback x Instruction interaction which occurred within eight clusters including dACC/mFC as well as bilateral insula/orbitofrontal and bilateral inferior parietal corticies (see Figure 2 and Table 1).

**Table 1:** Regions showing significant differences in the 4 (Feedback) ×3 (Instruction) Interaction.

		Coordinates			
Region	(*k*)	x	y	z	*F*-score
dACC/mFC	1037	9	9	60	22.75
Insula/OFC	508	42	15	−6	19.79
Insula/OFC	537	−39	12	0	16.21
Inferior Parietal Cortex	189	−57	−42	36	13.50
Inferior Parietal Cortex	152	60	−39	33	11.12
Inferior Frontal Cortex	109	48	0	45	10.31
Inferior Frontal Cortex	64	−42	−3	42	7.60
Precuneus	36	−36	−78	36	7.64

*Note*: All clusters met cluster-corrected thresholding of *p*<.05, FWE. dACC/mFC cluster spanned across both study ROIs.

Dissection of this interaction followed *a priori* hypotheses, and thus separately evaluated activity following *Informed-Inaccurate* feedback in each of the *Watch, Increase* and *Decrease* conditions.

### Baseline neural response to presented feedback

**Figure 3:**
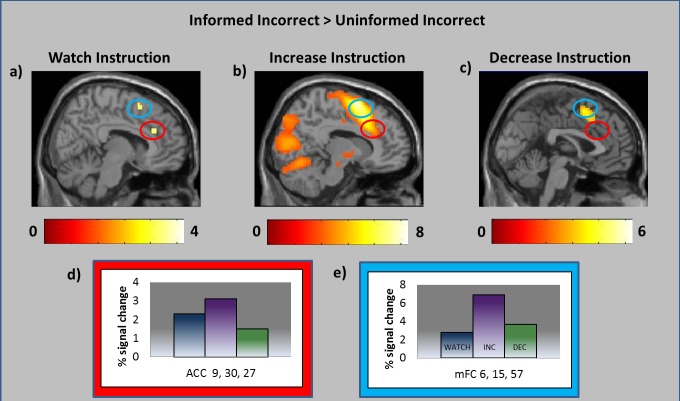
dACC/mFC activity in response to *Informative-Inaccurate* feedback in each of the *Watch* (Figure 3a), *Increase* (Figure 3b), and *Decrease* (Figure 3c) conditions. Note that dACC activity increased in the *Increase* condition, and decreased in the *Decrease* condition, compared to the *Watch* condition (Figure 3d), while mFC activity increased in both *Increase* (significantly) and *Decrease* (nonsignificantly) conditions (Figure 3e).

Participants' baseline responses to error feedback were investigated by evaluating activity following *Informed-Inaccurate* feedback in the *Watch* condition. A preliminary oneway ANOVA with Feedback as a within subject variable revealed no significant clusters; however, a planned comparison of the *Informed-Inaccurate > Uninformed-Inaccurate* contrast revealed significant peaks of activity within both dACC and mFC ROIs, as well as within left insula (see Figure 3a and Table 2). A similar comparison of the *Informed-Inaccurate > Informed-Accurate* contrast revealed no significant effects.

**Table 2:** Regions showing increased activity to Informed Inaccurate versus Uninformed Inaccurate feedback during Watch instructions.

		Coordinates			
Region	(*k*)	x	y	z	*t*-score
Dorsal Anterior Cingulate Cortex	6	9	30	27	2.72^*^
Middle Frontal Cortex	17	−33	33	42	2.88^*^
Insula	106	−39	15	6	3.34

*Note*: Whole-brain activity met cluster-corrected thresholding of *p*<.05, FWE.

Activity in *a priori* ROIs denoted with a * thresholded at *p*<.05, FWE-svc.

#### Efficacy of voluntarily-initiated increases in neural response to error feedback

Initial evaluation of participants' neural responses in the *Increase* condition were evaluated via oneway ANOVA, with Feedback as a within-subject variable. This analysis identified significant differences within several clusters, including a large cluster spanning across both dACC and mFC regions (*F* = 20.85, *k* = 8751). Subsequent planned comparisons indicated that this dACC/mFC cluster was enhanced following *Informed-Inaccurate* feedback compared to both *Uninformed-Inaccurate* (see Figure 3b and Table S1) and *Informed-Accurate* (see Figure S3a) feedback. To directly test for evidence of significant up-modulation, we compared neural activity following *Informed-Inaccurate* feedback in both the *Increase* and *Watch* conditions. This analysis indicated that participants showed significantly greater dACC/mFC response in the *Increase* condition, indicative of a successfully upregulated error-related response (see Figures 3d and 3e, which display percent signal change graphs for the dACC and mFC ROIs, respectively). Additional regions showing increased response in the *Increase* condition included supplementary motor area, bilateral insula, and bilateral inferior frontal cortex (see Table 3 for complete listing of all activated regions).

**Table 3:** Regions showing differential activity to Informed-Inaccurate feedback in the Increase and Decrease conditions compared to the Watch condition.

		Coordinates			
Region	*k*	x	y	z	*t*-score
*Increase > Watch*					
Activity spans: dACC/mFC/SMA	733	6	0	66	5.56*
Activity spans: Insula/iFC/Putamen	338	42	9	−3	4.98
Activity spans: Insula/iFC/sTC/Putamen	345	−39	6	0	4.44
Activity spans: mTC/smC	217	45	−45	6	4.25
Precentral Cortex	143	42	−6	45	4.57
Precentral Cortex	75	−48	−3	39	3.49
Supramarginal Cortex	123	−63	−39	27	3.77
*Increase <Watch*					
Activity spans: iPC/sPC	206	48	−69	42	4.72
Superior Frontal Cortex	176	24	24	57	4.29
*Decrease <Watch*					
Insula	37	45	15	−9	3.16
dACC	-	12	39	30	2.62*
*Decrease > Watch*					
Mid Frontal Cortex	145	−27	24	48	4.78
Activity spans Pre-/Post-central Cortex	446	−36	−18	57	5.22
Activity spans Anterior Insula/sTC	577	−36	−24	21	4.69
Angular Gyrus	165	45	−72	42	4.53
Lingual/Vermis/Precuneus	251	6	−54	3	4.12

*Note*: dACC  =  dorsal anterior cingulate cortex, mFC  =  medial frontal cortex, SMA  =  supplementary motor region, iFC  =  inferior frontal cortex, sTC  =  superior temporal cortex, iPC  =  inferior parietal cortex, smC  =  supramarginal cortex, sPC  =  superior parietal cortex. Whole-brain activity met cluster-corrected thresholding of *p*<.05, FWE.

Activity in *a priori* ROIs denoted with a * thresholded at *p*<.05, FWE-svc.

#### Efficacy of voluntarily-initiated decreases in neural response to error feedback

Evaluation of neural responses in the *Decrease* condition followed a similar course. Initial oneway ANOVA, with Feedback as a within-subject variable, revealed significant differences within several clusters, including a large cluster within mFC that extended somewhat into dACC (*F* = 8.18, *k* = 247, *p*<.05, FWE). Subsequent planned comparisons indicated that the dACC/mFC cluster was enhanced following *Informed-Inaccurate* feedback compared to both *Uninformed-Inaccurate* (see Figure 3c and Table S2) and *Informed-Accurate* (see Figure S3b) feedback. To directly test for evidence of significant down-modulation, we compared neural activity following *Informed-Inaccurate* feedback in both the *Decrease* and *Watch* conditions. Activity within the dACC, but not the mFC, trended towards a significant decrease in the *Decrease* condition (see Figure 3d and 3e for percent signal change estimates), suggesting that participants held a similar (albeit less robust) capacity to voluntarily down-regulate error-related neural responses following receipt of negative feedback.

#### Consideration of Neural Responses Underlying the Initiation of Effortful Control

**Figure 4:**
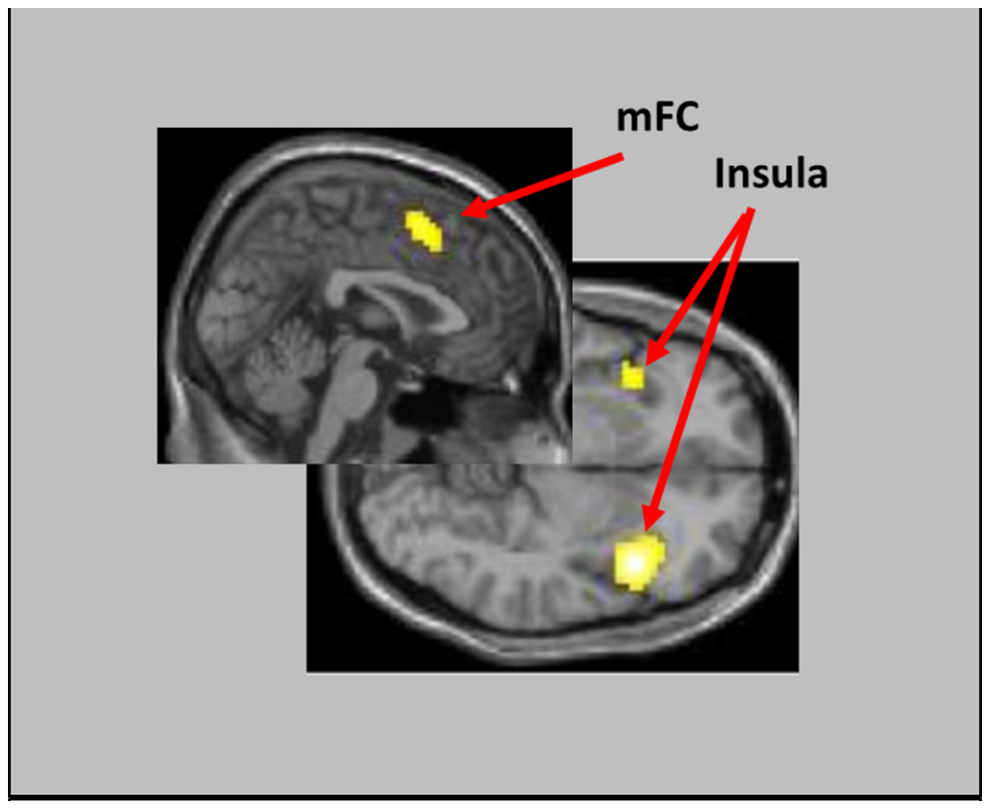
Regions significant in the conjunction analysis between Informed-Inaccurate > Uninformed-Inaccurate trials in both *Increase* and *Decrease* instruction conditions.

It is unlikely that neural changes observed in the *Increase* and *Decrease* conditions represented only participants' modulated responses. Rather, we may anticipate that attempts to voluntary modulate this response would necessarily also invoke neural resources underlying the utilization of effortful control strategies. Dissection of neural activity underlying the generation versus modulation of error-related responses is difficult, as the neural systems underlying generation versus modulation are likely to show considerable overlap [29]. However, while regions involved in the generation of error-related responses were expected to vary in the direction of the instructed modulation (as participants' dACC response did), we may expect regions underlying effortful control processes to show increased activity in both *Increase* and *Decrease* conditions. Consistent with this hypothesis, we identified several regions including middle frontal cortex, as well as left insular cortex, which showed enhanced responses in both *Increase* and *Decrease* conditions compared to the *Watch* condition. Moreover, a conjunction analysis of *Increase > Watch* and *Decrease > Watch* contrasts identified several clusters within mFC, insula and inferior frontal corticies that reached family-wise significance thresholds (see Figure 4). Importantly, however, directly contrasting the *Increase* and *Decrease* contrasts indicated that both dACC and mFC activity were greater in the *Increase* condition compared to the *Decrease* condition (see Table 4). Thus, we see evidence of successful modulation of dACC/mFC response, even under the backdrop of top-down resource recruitment.

**Table:** **Table 4.** Regions showing differential activity to Informed-Inaccurate feedback in the Increase compared to the Decrease condition.

		Coordinates			
Region	*k*	x	y	z	*t*-score
*Increase> Decrease*					
Activity spans: dACC/mFC/SFC	665	21	−30	66	5.51
		0	−6	48	4.89
		0	−9	66	5.22
Superior Frontal Cortex	105	−18	−30	60	4.60
Superior Temporal Cortex	169	39	−33	15	4.67
Superior Temporal Cortex	211	−48	−27	12	4.57
Superior Temporal Cortex	25	−60	−12	9	4.08
Pre/Post Central Cortex	152	−42	−15	45	5.04
Precentral Cortex	44	45	−12	42	4.27
Calcarine	179	−24	−63	9	4.25
Calcarine	28	27	−57	3	4.17
Insula	19	−33	6	3	4.07
*Increase <Decrease*	None				

*Note*: dACC  =  dorsal anterior cingulate cortex, mFC  =  medial frontal cortex, sFC  =  superior frontal cortex. Whole-brain activity met cluster-corrected thresholding of *p*<.05, FWE. Activity in *a priori* ROIs denoted with a * thresholded at *p*<.05, FWE-svc.

#### Voluntarily-induced modulation and post-feedback performance adjustments

**Figure 5:**
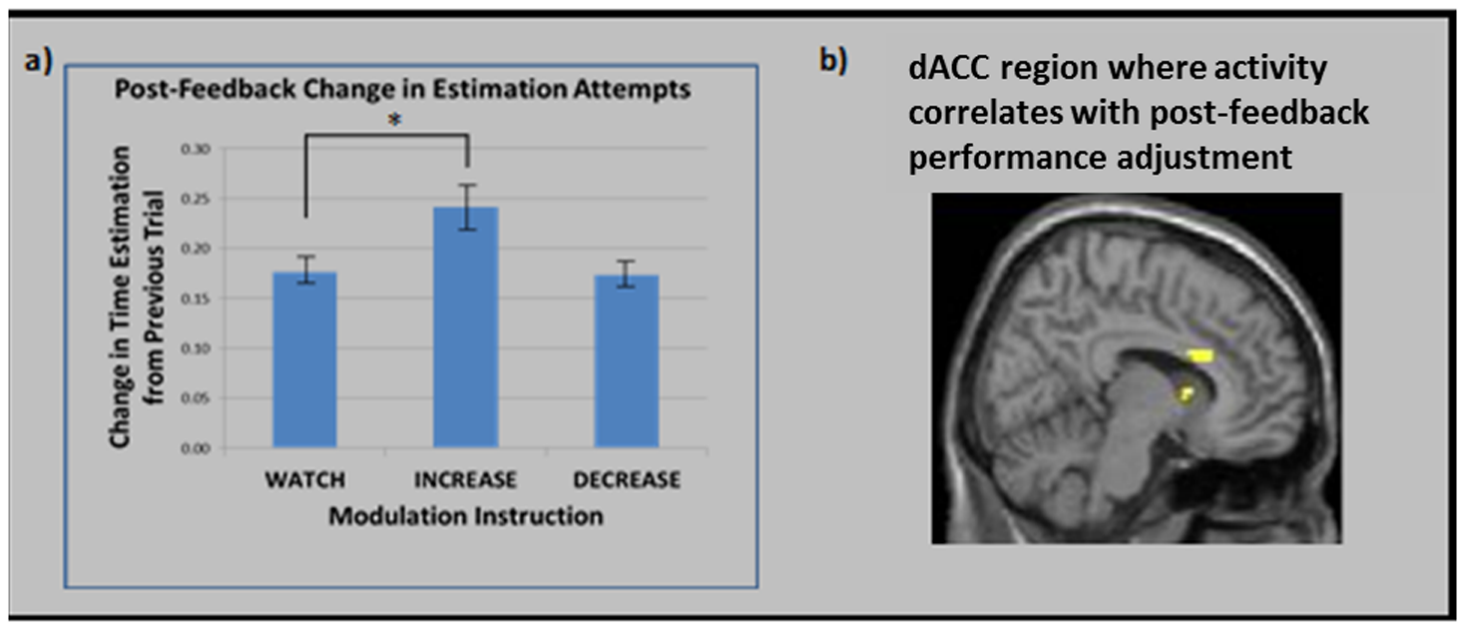
a) The degree to which participants altered their inaccurate time estimates following *Informed-Inaccurate* feedback in each of the *Watch, Increase* and *Decrease* modulation conditions. b) Region of dACC that correlated positively with magnitude of post-feedback change in time estimation in the *Watch* and *Increase*, but not the *Decrease*, modulation condition.

Figure 5a displays participants' estimation change scores following *Informed-Inaccurate* feedback in each of the *Watch, Increase* and *Decrease* conditions. Confirming hypotheses, participants showed higher change scores following *Informed-Inaccurate* feedback in the *Increase* condition compared to each of the *Watch* (*t* = 3.65, *p*<.05) and *Decrease* (*t* = 3.53, *p*<.05) conditions, which did not differ from each other (*p* >.10). Thus, participants showed greater post-feedback performance adjustment following inaccurate feedback when they were instructed to increase their neural response to that feedback, but did not show reduced post-feedback performance adjustments when instructed to decrease that neural response.

We also conducted a parametric modulation analysis in SPM5, whereby participants' trial-by-trial estimation change scores (see method section for relevant calculations) were entered as a parametric modulator locked to the presentation of the feedback stimulus. This analysis thus afforded a consideration of neural activity that correlated with the extent to which participants altered their estimation on the trial following receipt of inaccurate feedback. As hypothesized, this analysis identified a dACC cluster (peak coordinate: *x* = 9, *y* = 18, *z* = 21) that extended into our dACC ROI, which significantly predicted participants' change scores across both *Watch* and *Increase* conditions (but not in the *Decrease* condition; see Figure 4b).

## Discussion

The present study was designed to evaluate the extent to which participants could undertake voluntary up- and/or down-modulation of error-related neural activity in response to the presentation of negative feedback. To this end, participants were asked to perform a simple time-estimation task under a standard *Watch* condition, and under *Increase* and *Decrease* conditions during which they were asked to intentionally enhance or reduce their brain's response following the presentation of the negative feedback. Despite being given no guidance as to how to accomplish this neuromodulation, participants showed considerable capacity for altering their neural responses in the instructed direction. Activity within a large cluster that spanned across hypothesized regions increased in the *Increase* condition; and a more specific sub-cluster within dACC trended towards a significant reduction in the *Decrease* condition. To our knowledge, this is the first study to demonstrate that neural activity underlying the processing of error-related information may be modulated through such voluntary efforts (but see [30] for evidence that one's emotional response to error can be regulated).

This finding may be interpreted within the context of a growing literature suggesting that humans may have the capacity to invoke substantive influence over the nature of their neural responses to specific stimulus types, including emotionally-valent pictures [15], [31], pain [32], stimuli that evoke craving [33], and during the processing of happy memories [34]. The ability to modulate neural activity to negative feedback may have particularly practical implications, however. The magnitude of this dACC response is believed to index the activity of a generalized error-monitoring system important for the adaptive selection and guidance of subsequent corrective behavior [6]–[8]. If this is true, then up-regulation of this dACC response could facilitate one's ability to learn through trial-and-error, and to manage goal-directed behavior in the face of changing environmental demands. The results of the present study support this hypothesis: magnitude of dACC response following negative feedback correlated with the extent to which participants adjusted their estimation attempts on the following trial (in the *Increase* and *Watch* conditions, at least). Moreover, estimation change scores correlated with dACC response across the *Watch* and *Increase* conditions, and were higher in the *Increase* condition compared to the *Watch* and *Decrease* conditions. While these results did not translate to the *Decrease* condition, they nonetheless suggest that participants' voluntary modulation of their neural response to the negative feedback also influenced the extent to which they adjusted their post-feedback behavior. The possibility that more prolonged neuromodulatory training could serve to facilitate operant learning proficiency is an intriguing possibility that future research could further consider (see [35]).

It is also relevant to note that differential responsivity to error has been shown characteristic of a variety of personality characteristics related to clinical and subclinical states. Individuals with low levels of inhibitory control, including those with substance abuse disorders and attention hyperactivity deficit disorder (ADHD) have, for instance, been characterized with a hypoactive dACC response to error-related information [36], [37]. Individuals characterized by heightened levels of anxiety have, in turn, been characterized with increased dACC response to error-related information [21], [38], The extent to which these individual differences would themselves influence the capacity to successfully undertake dACC neuromodulation to error remains an open question, but one that future research may do well to consider. It should also be noted, however, that participants in the present study showed dACC reductions in the *Decrease* condition that only reached trend levels of significance – while this may be the result of the small sample size of the present study, it also may deem consideration of therapeutic benefits of dACC down-regulation somewhat premature.

It is important to note that our two primary regions of interest showed somewhat different patterns of activity across the three instruction conditions. Activity in dACC showed the hypothesized pattern: activity increased in the *Increase* condition, and decreased in the *Decrease* condition, compared to the *Watch* condition. However, activity in mFC, as well as in insular and inferior frontal regions, showed significant increases in both the *Increase* and *Decrease* conditions. A simple interpretation of this data could be that participants were capable of modulating their dACC response to the presentation of negative feedback, but that this capacity did not extend to adjacent mFC regions. However, such interpretation would ignore the extent to which participants' modulation attempts themselves required the recruitment of frontoparietal resources towards the initiation of top-down effortful control [39], [40]. Indeed, we may anticipate increased control-related recruitment in both the *Increase* and *Decrease* conditions, as participants attempt to modulate their neural reactivity in the instructed direction. In this context, the increased mFC activity seen across *Increase* and *Decrease* conditions may not imply decreased modulation capacity, but rather increased recruitment of regulatory resources. To separate activity associated with each process, we undertook a direct comparison of post-error feedback activity in the *Increase* and *Decrease* conditions (both of which should have required similar recruitment of effortful control processes). This contrast identified increased activity in both dACC and mFC in the *Increase* condition. We interpret this as evidence of successful modulation of dACC/mFC response, even after parsing activity associated with the initiation of resource-intensive top-down control processes.

This study is not without its limitations. First, our sample size is relatively modest, which may have challenged our ability to identify small- or medium-sized effects. This may be particularly relevant when interpreting the results from the *Decrease* condition, where dACC reductions only reached trend significance levels. Future research would do well to replicate this effect within a larger sample, at which point more conclusive evidence for the capacity to down-modulate error-related responses may be acquired. Second, we once again acknowledge the challenges associated with trying to distinguish between generative versus regulatory processes. This is an oft-acknowledged challenge [17] that characterizes the majority of emotion-regulation work, and the present study is no exception. The time-estimation paradigm was not designed to explicitly leverage the ability to distinguish generative versus regulatory processing; however, direct contrast of the *Increase* and *Decrease* conditions provided some useful insights. Future work specifically designed to isolate the heavily overlapping processes would greatly benefit the field.

To summarize, our results expand on work undertaken within the "emotion regulation" literature, and demonstrate that individuals have the capacity for voluntary modulation of neural activity underlying a more cognitively-mediated error-monitoring process. This synthesizes well with growing work indicating the plasticity of neural structure and function, and highlights the fact that such plasticity is not necessarily reliant on emerging high-tech methodologies such as TMS, TDCS or rt-fMRI. It is frequently assumed that both automatic and controlled emotion regulation strategies serve adaptive (and/or maladaptive) self-regulatory functions; the extent to which cognitive regulation strategies also serve such functions has received less, and perhaps less-than-warranted, attention.

## Supporting Information

Figure S1 **Main effect of feedback.**
(TIF)

Figure S2 **Main effect of instruction.**
(TIF)

Figure S3 a) Significant clusters within the Informed-Inaccurate > Informed-Accurate contrast in the *Increase* condition; b) Significant clusters within the Informed-Inaccurate > Informed-Accurate in the *Decrease* condition.
(TIF)

Table S1 **Regions showing increased activity to Informed-Inaccurate compared to Uninformed-Inaccurate feedback in the Increase instruction condition.**
(DOCX)

Table S2 **Regions showing increased activity to Informed-Inaccurate compared to Uninformed-Inaccurate feedback in the Decrease instruction condition.**
(DOCX)
