# Supplementary figures and images for: Voluntary Modulation of Anterior Cingulate Response to Negative Feedback
Source: PLoS One. 2014 Nov 6;9(11):e107322. doi: 10.1371/journal.pone.0107322 (PMC4222862; doi:10.1371/journal.pone.0107322)

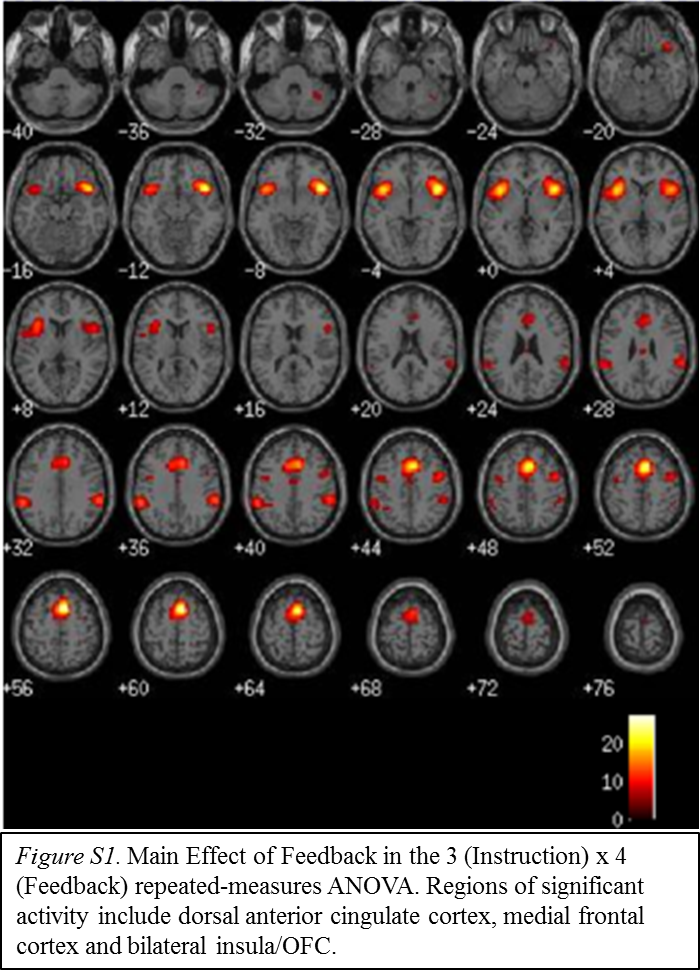

Supplement: Figure S1 — Main effect of feedback. (TIF) [file pone.0107322.s001.tif]

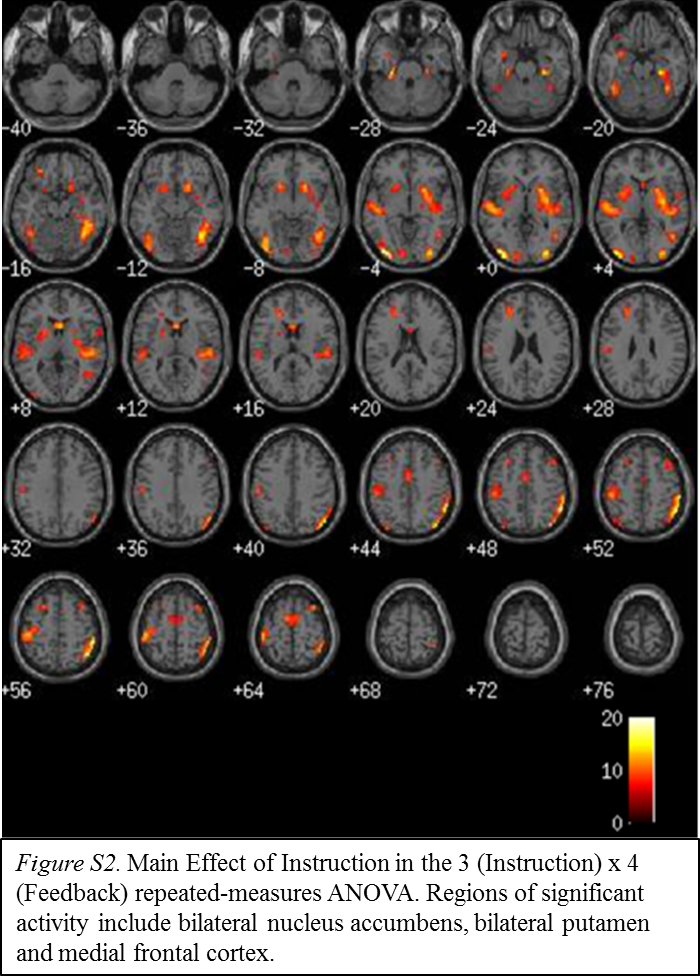

Supplement: Figure S2 — Main effect of instruction. (TIF) [file pone.0107322.s002.tif]

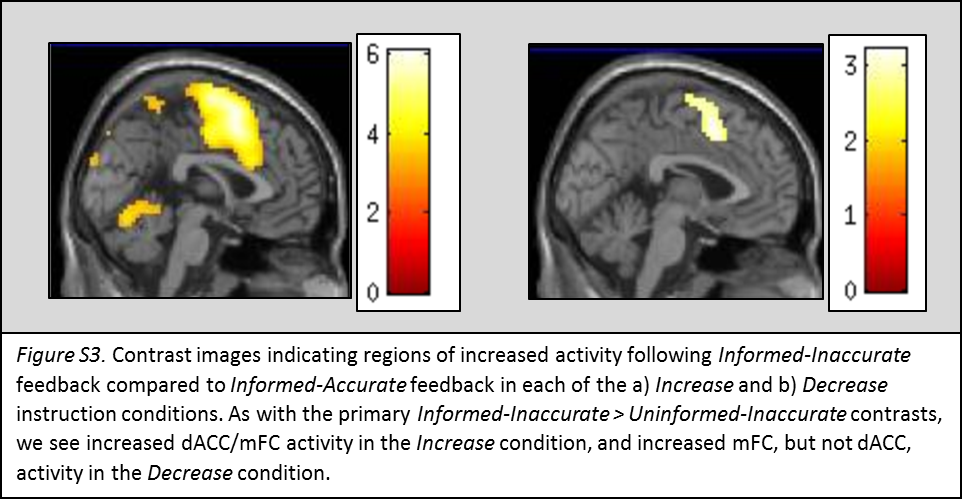

Supplement: Figure S3 — a) Significant clusters within the Informed-Inaccurate > Informed-Accurate contrast in the Increase condition; b) Significant clusters within the Informed-Inaccurate > Informed-Accurate in the Decrease condition. (TIF) [file pone.0107322.s003.tif]
