# Supplementary material for: Voluntary Modulation of Anterior Cingulate Response to Negative Feedback
Source: PLoS One. 2014 Nov 6;9(11):e107322. doi: 10.1371/journal.pone.0107322 (PMC4222862; doi:10.1371/journal.pone.0107322)
Supplement: Table S1 — Regions showing increased activity to Informed-Inaccurate compared to Uninformed-Inaccurate feedback in the Increase instruction condition. (DOCX) [file pone.0107322.s004.docx]

Table S1

*Regions showing increased activity to Informed-Inaccurate compared to Uninformed-Inaccurate feedback in the Increase instruction condition.*

|  |  | Coordinates | | |  |
| --- | --- | --- | --- | --- | --- |
| Region | *k* | x | y | z | *t*-score |
| Activity spans: dACC, mFC, sFC, SMA | 1697 | 6 | 9 | 63 | 7.69^*^ |
| Dorsomedial Frontal Cortex | 48 | 30 | 48 | 24 | 3.86 |
| Dorsolateral Frontal Cortex | 54 | -30 | 45 | 21 | 3.53 |
|  |  |  |  |  |  |
| Activity spans: Insula, iFC, sTC | 659 | 42 | 15 | -6 | 6.31 |
| Activity spans: Insula, iFC | 1165 | -39 | 9 | 0 | 6.04 |
|  |  |  |  |  |  |
| Activity spans: iPC, SMC | 559 | -54 | -39 | 33 | 5.66 |
| Activity spans: Precentral Cortex | 348 | 45 | -3 | 42 | 5.40 |
| Activity spans: mTC, smC | 528 | 48 | -33 | -6 | 4.82 |
| Postcentral Cortex | 61 | 21 | -27 | 63 | 4.23 |
| Postcentral Cortex | 27 | -21 | -30 | 68 | 3.40 |
| Inferior Parietal Cortex | 22 | -30 | -48 | 48 | 3.36 |
|  |  |  |  |  |  |
| Occipital Cortex | 465 | 15 | -81 | 12 | 4.08 |
| Precuneus | 98 | 18 | -72 | 39 | 3.84 |
| Precuneus | 245 | -6 | -75 | 45 | 3.83 |
| Cerebellum | 280 | 36 | -57 | -30 | 4.17 |
| Cerebellum | 63 | -27 | -63 | -24 | 3.65 |

*Note:* dACC = dorsal anterior cingulate cortex, mFC = medial frontal cortex, sFC = superior frontal cortex, SMA = supplementary motor region, iFC = inferior frontal cortex, sTC = superior temporal cortex, iPC = inferior parietal cortex, smC = supramarginal cortex. Whole-brain activity thresholded at *p* < .001, uncorrected. Activity in *a priori* ROIs denoted with a * thresholded at *p* < .05, FWE-svc.
