# Supplementary material for: Voluntary Modulation of Anterior Cingulate Response to Negative Feedback
Source: PLoS One. 2014 Nov 6;9(11):e107322. doi: 10.1371/journal.pone.0107322 (PMC4222862; doi:10.1371/journal.pone.0107322)
Supplement: Table S2 — Regions showing increased activity to Informed-Inaccurate compared to Uninformed-Inaccurate feedback in the Decrease instruction condition. (DOCX) [file pone.0107322.s005.docx]

Table S2

*Regions showing increased activity to Informed-Inaccurate compared to Uninformed-Inaccurate feedback in the Decrease instruction condition.*

|  |  | Coordinates | | |  |
| --- | --- | --- | --- | --- | --- |
| Region | *k* | x | y | z | *t*-score |
|  |  |  |  |  |  |
| Medial Frontal Cortex | 260 | 6 | 12 | 54 | 4.16 |
|  |  |  |  |  |  |
| Insula | 401 | 45 | 15 | -6 | 5.30 |
| Insula | 141 | -39 | 15 | -3 | 3.77 |
|  |  |  |  |  |  |
| Inferior Parietal Cortex | 128 | 60 | -39 | 33 | 4.34 |
| Inferior Parietal Cortex | 76 | -57 | -45 | 39 | 3.65 |

*Note:* Whole-brain activity met cluster-corrected thresholding of *p* < .05, FWE.

Activity in *a priori* ROIs denoted with a * thresholded at *p* < .05, FWE-svc.
